# Procreative Non-Maleficence: A South African Human Rights Perspective on Heritable Human Genome Editing

**DOI:** 10.1089/crispr.2019.0036

**Published:** 2020-02-17

**Authors:** Donrich Thaldar, Bonginkosi Shozi

**Affiliations:** School of Law, University of KwaZulu-Natal, Durban, South Africa.

## Abstract

If the safety and efficacy issues relating to heritable genome editing can be resolved, how should liberal democratic societies regulate the use of this technology by prospective parents who wish to effect edits to the genomes of their prospective children? We suggest that recent developments in South African law can be useful in this regard. The country's apex court recently recognized as a legal principle that the scope of possible reproductive decisions that parents may make when using new reproductive technologies excludes decisions that will cause harm to the prospective child—the principle of procreative non-maleficence. We suggest that the principle of procreative non-maleficence provides a mechanism for striking an equitable balance between two competing interests that are given legal recognition in most liberal democracies: the reproductive rights of prospective parents and the state's duty to protect child welfare.

## Introduction

The news of the latest innovation in genome editing technology, prime editing, has underscored the reality that we live in a time when the possibility of safe and efficacious heritable genome editing may be within reach, and perhaps is closer than initially thought.^1^ Prime editing advances on existing CRISPR genome editing techniques in that it allows for precise alterations to DNA without double-strand breaks. In addition to being more precise, prime editing has the potential to mitigate (and perhaps eliminate) unintended adverse outcomes of genome editing such as off-target effects.^2^

It is undoubtedly too soon to herald prime editing as the future of human genome editing. However, even at these early stages, it is noteworthy, as it highlights that the technical challenges to heritable gene editing can be overcome. The restrictive regulation of heritable genome editing, in those few states that regulate it, has largely been justified on the grounds that heritable genome editing is too unsafe for clinical application.^3^ Given that safe and efficacious heritable genome editing may be on the horizon, it is time for policy makers to give serious consideration to the legal dimensions of gene editing beyond these technical issues.

The relevance of human rights to heritable genome editing is a topic that has often been absent in the global debate on heritable genome editing using CRISPR-Cas9. Of particular import in this regard are the reproductive rights of the prospective parents who would be choosing to have a genetically modified child. Of the many ethics statements on genome editing to emerge in recent years, the only one to give significant regard to the potential role to be played by reproductive rights is the report by the Nuffield Council on Bioethics.^4^ This report notes that the use of CRISPR-Cas9 intersects with the high premium that modern liberal democracies give to the need to respect the reproductive goals of people seeking to become parents. Indeed, in states such as South Africa (SA) that give legal protection to the freedom of parents to make decisions concerning reproduction, including choosing to use new reproductive technologies, the use of CRISPR-Cas9 for heritable genome editing would be something to which prospective parents are *prima facie* entitled.

That said, heritable genome editing potentially relates not only to the rights of prospective parents but also to the rights of the children who may be born with genetically altered genomes. Scholars such as Knoppers and Kleiderman have pointed out that given that heritable genome editing can impact the health and well-being of the prospective child, consideration must be given to the principle that in all matters concerning the child, the best interests of the child should be paramount—commonly referred to as the child welfare principle.^5^ Most liberal democracies recognize the child welfare principle in their domestic law or are party to international agreements that enjoin them to give effect to it, such as the United Nations Convention on the Rights of the Child.^6^

While in one sense heritable genome editing may be viewed as promoting the best interests of the child when used for therapeutic purposes, it might also be viewed as potentially compromising the welfare of the prospective child. For instance, proponents of a moratorium on heritable genome editing have raised, among their many concerns, that “children with edited DNA could be affected in detrimental ways,” such as by experiencing psychological harm as a result of knowing that they were born with altered genomes.^7^ As such, the stage is set for potential conflict between reproductive rights on the one hand and the child welfare principle on the other.

Given this potential conflict, a principle applied in a recent SA case, *AB* v. *Minister of Social Development*,^8^ may prove a useful guide to policy makers in other liberal democracies that are plotting regulatory pathways for heritable genome editing. In this article, we describe the genesis of this principle in SA law, analyze the main critique of the principle, and illustrate how this principle may be used in determining what uses of heritable genome editing technology ought to be permissible in a liberal democracy.

## The *AB* Majority Judgment and the Principle of Procreative Non-Maleficence

The child welfare principle first emerged in SA in common law and was applied by courts when determining familial disputes over custody and access to children.^9^ Under the influence of growing international emphasis on protecting the interests of children as a vulnerable group through provisions committing states to make the welfare of children paramount, the child welfare principle was included in the final draft of the SA Constitution in 1996 in a form very similar to those seen in prominent international instruments.^10^ Since then, the child welfare principle has expanded beyond its usual domain of family law, and has most recently featured prominently in reproductive law. The way in which the child welfare principle applies in this context was illustrated in the *AB* case.

The *AB* case concerned surrogate motherhood and the use of donor gametes.^8^ In SA law, a woman who undergoes *in vitro* fertilization (IVF) has the right to use male and female donor gametes, irrespective of whether it is medically indicated, and, further, has the right to select a gamete donor based on *inter alia* the donor's characteristics, such as the donor's educational level, race, and skin tone.^11^ However, and seemingly paradoxically, SA statutory law requires a commissioning parent in the context of surrogacy to use his or her own gametes (“the own-gametes requirement”).^12^ The applicant in the *AB* case was an infertile woman who intended to become a mother through a surrogacy arrangement. However, given that she could not contribute her own eggs for the conception of the surrogate child, she was legally banned from using surrogacy. The applicant challenged the constitutionality of the own-gametes requirement in court. She argued that the own-gametes requirement infringed on several of her constitutional rights, including her reproductive rights.

The Minister of Social Development, who is the cabinet-level official responsible for administrating the impugned statute, opposed the application, relying on the child welfare principle. The Minister argued that it is in the best interests of children that they should know their genetic origins. In many traditional African cultures, knowing one's genetic origins is essential for clan membership and hence for a child's self-identity.^13^

At this stage, however, there was no child in existence. Therefore, what the Minister was effectively proposing was that the child welfare principle should apply to the *prospective* child. Importantly, the applicant did not take issue with the application of the child welfare principle to the prospective child. Instead, she adopted a strategy of proving through expert evidence by psychologists that not knowing one's genetic origins is unlikely to impact negatively on one's overall psychological well-being and therefore does not constitute harm to the prospective child.

The *AB* case eventually reached SA's apex court, the Constitutional Court. The 11 justices of the Constitutional Court bench were sharply divided, and they handed down a majority and minority judgment. The majority of the Constitutional Court held that the court must indeed protect the best interests of the *prospective* child. The tacit principle underlying the majority judgment can be articulated as follows:

The scope of possible reproductive decisions that prospective parents may take, at least in the context of artificial reproduction, should be legally limited to exclude decisions that will cause harm to the prospective child.

We suggest that this principle can aptly be referred to as the principle of “procreative non-maleficence.” The idea of procreative non-maleficence is not new, and various versions of it have been proposed in the ethics literature.^14–16^ In essence, it is simply the application of John Stuart Mill's classic harm principle to the emerging new area of artificial reproduction.^17^ However, the *AB* judgment represents the first time this principle has been utilized in litigation in an attempt to strike a balance between the rights of prospective parents and prospective children.

## Harm in the Law

While the *AB* minority frequently used the word “harm”—also with reference to the majority's reasoning—the majority itself used “risk” to the (prospective) child. In the context, we suggest “risk” to the prospective child is best understood as “risk of harm” to the prospective child. But what exactly will constitute *harm* to the prospective child? The *AB* judgment did not explore this question in any detail.

Mill made it clear that harm does not include mere offense to people's sensibilities. To constitute harm, an act must violate a right or an important interest. However, at least since harm's rise to prominence in Mill's *On Liberty*, its exact meaning has been a point of contention in philosophy.^18,19^ In law, where the concept of harm has come to play an important role in both criminal law and the law of civil wrongs (delict or tort), the concept is ever evolving to reflect society's *boni mores*.^20,21^ This evolving nature of the exact parameters of harm is not a weakness but rather a strength, as it avoids the ossification of the concept in one point in time. Given that the objective of the principle of procreative non-maleficence is to build a conceptual bridge between protecting the best interests of *existing* children and protecting the best interests of *prospective* children, an equivalent conceptual bridge should be constructed in our understanding of harm to the prospective child. This can be formulated as follows:

If a reproductive decision by a prospective parent is likely to have an effect on the prospective child that would constitute either a civil or criminal wrong in law if caused by an act by a parent toward an existing child, such reproductive decision would constitute harm to the prospective child.

But does the idea of harming a prospective child not create a legal fiction that a prospective child is a person with rights? Not necessarily. One must remember that the wrongful act and the subsequent harm can take place at different points in time. As a result, the person who suffers the harm does not need to be in existence when the wrongful act occurs, only when the harm occurs. For instance, a child who is born with disability (the harm) due to a car accident (the wrongful act) that took place during the pregnancy—before the child's existence as a person in law—can hold the negligent driver who caused the accident and hence the disability liable for a civil wrong.^22^ The concept of harm to a prospective child is therefore properly understood as an anticipated future event: harm that will materialize and affect the prospective child if and only after he or she comes into existence.

## Postscript to the *AB* Majority Judgment

Unfortunately, when applying the principle of procreative non-maleficence to the facts of the case, the *AB* majority judgment floundered. It turned its back on the evidence presented by the applicant, and relied on unsubstantiated value judgments regarding the significance of knowing one's genetic origins to children's welfare.^23^ The majority held that not knowing the identity of one's genetic parents constitutes “risk” to the prospective child, and hence ruled in favour of the Minister and upheld the statutory own-gametes requirement. We suggest that the majority erred in the way it applied the principle of procreative non-maleficence. This does not, however, detract from the importance of the establishment of procreative non-maleficence as a general legal principle that can provide useful guidance in the near future when heritable genome editing may become a safe and efficient reproductive choice that intended parents can make. That said, it does underscore the importance of mooring one's understanding of harm to the *terra firma* of evidence and of legal precedent regarding harm, which can be accomplished using our proposed existing-child-analogy test.

## Against the Principle of Procreative Non-Maleficence: The Nonidentity Problem—*Ex Parte KAF*

Can a parent ever harm a child by bringing that child into existence, if the only alternative of such harm would have been the child's non-existence? This question lies at the core of the chief criticism of the principle of procreative non-maleficence: that it fails as a principle because no choices made before a person comes into existence can be construed as harm if, without those choices being made, that person would not have existed. The *AB* minority raised this question, commonly referred to as the non-identity problem, made famous in the work of Derek Parfit.^24^ A common response to the non-identity problem is that existence must have a negative quality for non-existence to be preferable. Another response to the non-identity problem is to navigate around it by postulating harm in the context of human reproduction as not being “person-affecting” (relating to the specific child-to-be-born) but instead being “others-affecting” in the sense that it affects the general well-being of people in society. Relying on this roundabout solution, Ben Saunders recently proposed the Principle of *generalized* procreative non-maleficence, entailing that there is a moral obligation on parents not to cause harm to other people through their reproductive choices.^16^ Although there is merit in considering the wider societal impact of reproductive choices, in the following paragraphs, we explore a head-on solution to the non-identity problem that focuses on harm to the prospective child.

In SA law, surrogacy agreements must be confirmed by the court before the parties may proceed with the artificial fertilization of the surrogate mother. In such a surrogacy agreement confirmation hearing, *Ex Parte KAF*,^25^ the Johannesburg High Court was confronted with a situation where the commissioning parents intended to use existing embryos—embryos that were originally intended for the commissioning mother's own attempts to fall pregnant through IVF. The question raised was whether it is incumbent upon the court to consider the best interests of these embryos as possible children to be born. The court answered this question with a clear no, and made a conceptual distinction between the mental construct of the prospective child and the physical embryos. The court held that “not one of these embryos can be legally equated with the child that is to be born,” and that “the embryos are merely the human biological material that may … give rise to the child that is to be born.” In other words, what the court was saying in *KAF* was that in legal terms, there is no continuity of identity between any particular *in vitro* embryo and the identity of the person who is born using that embryo.

In light of the *KAF* judgment, consider the following hypothetical scenario of decision making in the context of artificial reproductive technologies. Suppose prospective parents have two *in vitro* embryos to choose from: embryo A appears healthy in all respects, while embryo B has a genetic condition that will likely cause serious disease. If the prospective parents choose to use embryo B, or knowingly allow such a choice, are they harming their prospective child? The answer suggested by the non-identity problem is no. The child that will result from Embryo B is no worse off than he or she might otherwise have been because the only alternative, namely the parents choosing embryo A, is that this specific child would not exist. This implies there are two distinct prospective children with different identities: one that will emanate from embryo A, and one that will emanate from embryo B. In other words, there is a continuity of identity between embryo and child. Clearly, this reasoning is incompatible with the *KAF* judgment, which held that not one of the embryos can be equated with the prospective child. Following on the *KAF* judgment, the identity of the child is continuous with the mental construct of the prospective child and not with the embryo. Similar to other significant choices that parents make for their child, such as in which culture and language community their child will be raised, parents can choose the human biological material—the embryo—that will develop into a physical body for the child. All of these choices will impact on the child's identity in the sense of being attributes, but none are definitive in the sense that they will change the child into a different child—at least not from a legal perspective. Accordingly, the non-identity problem entails a false dilemma. The true alternative to giving the prospective child genes that will likely cause serious disease (choosing embryo B) is to give the (same) prospective child genes that will likely lead to a normal, healthy life (choosing embryo A).

It follows that the *AB* minority's argument against the principle of procreative non-maleficence fails to convince.

## Conclusion

From our discussion, it should be clear that the principle of procreative non-maleficence is not a zero-level threshold, meaning that the prospective child's existence must merely be better than non-existence. The non-maleficence threshold is higher. It applies the same minimum standard that generally applies to the legal relationship between parents and their *existing* children to the legal relationship between parents and their *prospective* children. At the same time, the principle of procreative non-maleficence is not a maximizing principle but a sufficing principle, best conceptualized as establishing a threshold or minimum standard. Setting minimum standards of conduct that parents must adhere to based on an evolving societal concept of harm is the way in which the law generally regulates the relationship between parents and their (existing) children. For instance, in most liberal democracies, parents are legally compelled to ensure that their children receive at least a basic education, but they are free to complement such basic education in ways that *they deem fit*. Not receiving at least a basic education is generally perceived as harm to the child, and can lead to criminal—and perhaps even civil—liability of the parents. Likewise, prospective parents should be free to use genome editing technology to design their prospective children's genetic makeup in any way that *they deem fit*—as long as they adhere to the minimum standard of no harm to the prospective child.
